# Fabrication of Composite Filaments with High Dielectric Permittivity for Fused Deposition 3D Printing

**DOI:** 10.3390/ma10101218

**Published:** 2017-10-23

**Authors:** Yingwei Wu, Dmitry Isakov, Patrick S. Grant

**Affiliations:** Department of Materials, University of Oxford, Parks Road, Oxford OX1 3PH, UK; yingwei.wu@materials.ox.ac.uk (Y.W.); patrick.grant@materials.ox.ac.uk (P.S.G.)

**Keywords:** dielectric composites, ceramic, 3D printing, additive manufacturing

## Abstract

Additive manufacturing of complex structures with spatially varying electromagnetic properties can enable new applications in high-technology sectors such as communications and sensors. This work presents the fabrication method as well as microstructural and dielectric characterization of bespoke composite filaments for fused deposition modeling (FDM) 3D printing of microwave devices with a high relative dielectric permittivity ϵ=11 in the GHz frequency range. The filament is composed of 32 vol % of ferroelectric barium titanate (BaTiO_3_) micro-particles in a polymeric acrylonitrile butadiene styrene (ABS) matrix. An ionic organic ester surfactant was added during formulation to enhance the compatibility between the polymer and the BaTiO_3_. To promote reproducible and robust printability of the fabricated filament, and to promote plasticity, dibutyl phthalate was additionally used. The combined effect of 1 wt % surfactant and 5 wt % plasticizer resulted in a uniform, many hundreds of meters, continuous filament of commercial quality capable of many hours of uninterrupted 3D printing. We demonstrate the feasibility of using the high dielectric constant filament for 3D printing through the fabrication of a range of optical devices. The approach herein may be used as a guide for the successful fabrication of many types of composite filament with varying functions for a broad range of applications.

## 1. Introduction

The development of multiple material 3D printing capability, enabling the fabrication of composite structures with a broad range of electromagnetic properties, will open up new possibilities for novel functional structures utilising the principles of transformation optics [1,2], smart microwave devices, and systems possessing meta-material features [3]. Available techniques for multiple material 3D printing are mostly limited to extrusion-based and photo-solidification approaches where the printing material matrix (usually a thermoset polymer or a photoactive resin) is mixed with active particles (e.g., colour dye [4,5], dielectric [6] and magnetic micro-particles [7,8], carbon nanofibers [9,10], wood fibers [11], etc.) that bring the desired functionality to the final composite printed component. 3D printing also allows for spatial variations in functionality, such as grading or anisotropy [3].

While a very wide range of composite feedstock materials for additive manufacturing are commercially available, there are restricted materials with specific properties that might be potentially used for the 3D printing of functional active devices, and the formulation of such materials and composites with robust and reproducible high-performance properties remains a challenge.

The stereolithography (SL) process with mixed fine ceramic powders suspended in a liquid photocurable resin has been used for many years for the fabrication of green parts with arbitrary final 3D shape [12]. Using photosensitive acrylic resin with piezoelectric micro-particles of 0.65Pb(Mg_1/3_Nb_2/3_)O_3_-0.35PbTiO_3_, and magnetite nanoparticles, SL also has been used for the fabrication of a green structure for piezoelectric [13] and magnetic flow sensors [8], respectively. The same method was utilised to demonstrate various 3D structures with excellent capacitive property and high dielectric constant using Ag-surface-coated nanoparticles of PbZr_*x*_Ti_1−*x*_O_3_ incorporated into the photocurable polymer solution [14]. An alumina/UV-cured-resin composite has also been used to demonstrate a conical insulating spacer with an effective dielectric constant of 6.5 at low frequency [15].

Leight et al. used a polycaprolactone thermoplastic matrix and magnetite nanoparticles to fabricate feedstock filament (although the volume fraction of magnetite was not reported) for fused deposition modelling (FDM) printing, and demonstrated a fully 3D-printed flow sensor [16]. A polycaprolactone matrix was also used for the fabrication of conductive filament using carbon black. The loading of carbon black within the polymer matrix was optimised (through tests to observe how the composites performed under extrusion through the printer nozzle) so as to be easily printable by a desktop FDM machine without modification [10] .

Additionally, bespoke composite filaments are also widely used for the 3D printing of structural composites. FDM with Cu/acrylonitrile butadiene styrene (ABS), and Fe/ABS composites of up to 40 vol % filler have been used to show significant improvements in ABS mechanical and thermal properties [17]. A 5 wt % tungsten–polycarbonate composite was fabricated as an FDM feedstock filament for space-based applications, and printed parts showed an improvement in X-ray radiation shielding [18]. Carbon nanotubes have been used to reinforce thermoplastic polyurethane filaments and to induce electrical conductivity [19,20,21]. The use of thermotropic liquid crystalline polymer fibrils resulted in the improvement of mechanical strength of polypropylene composite feedstock [22].

Magnetized alumina particles’ orientation in a polymer matrix can be effectively controlled by using a magnetically-assisted 3D printing platform [23]. This approach allows the fabrication of highly anisotropic structures. For example, a large anisotropy in thermal conductivity was contrived in a 3D-printed 4.3 vol % graphite/ABS composite due to the as-printed alignment of graphite flakes [24]. Duigou et al. also showed that wood fibre-reinforced filaments could be used to achieve mechanical anisotropy in 3D-printed composite materials [11]. In Ref [25] it was reported that a cellulose blend with polylactic acid (PLA) resulted in increased crystallinity of PLA and therefore showed an increase in storage modulus.

Among the many additive manufacturing techniques, FDM remains the most popular due to its low cost, flexibility, and open community RepRap [26] society support. However, the supply of composite filaments, for example of functional ceramics in a printable polymer matrix for FDM usage, is mainly limited by the brittleness of the resulting composite filament as the fraction of the functional ceramic is increased, which leads to unreliable printing. For example, in [6] the dielectric characterisation of BaTiO_3_/ABS filaments was presented, but loadings above 70 wt % of BaTiO_3_ were not investigated, as the filament became too brittle to be readily printed, causing snapping during spooling and/or feeding. This maximum loading threshold may be varied depending on the specific inorganic oxides material [3], but was given as approximately 27 vol % for ferroelectric BaTiO_3_ micro-particles possessing the highest relative dielectric permittivity ϵ ~ 500 [27] at microwave frequencies.

The objective of this work is to study and improve the binding mechanism in composite polymer ABS and BaTiO_3_ ceramic micro-particles in order to improve the fabrication route of a continuous uniform filament possessing high dielectric permittivity. The use of an optimal amount of hydroxylbensoic ester derivative binder (octyl gallate, C_15_H_22_O_5_) and a plasticiser (dibutyl phthalate, C_18_H_22_O_4_) is shown to facilitate an increase in the maximum volume fraction of filler particles and simultaneously decreases the stiffness and brittleness of the filament. This results in an increase in the relative dielectric permittivity of the final composite of approximately 36% over previous work, and produces long lengths of filament for FDM of commercial quality. These filaments allowed many hours of uninterrupted 3D printing of complex structures with spatially varying electromagnetic properties for transformation electromagnetic or smart microwave devices.

## 2. Results and Discussion

### 2.1. Effect of Surfactant

Residual air voids spaces (pores) in extruded ceramic–polymer composite filaments for FDM are the most serious defects, and limit the bend and/or tensile properties due to reduced load carrying capability and stress concentration effects. Here, we use a surfactant to act as a bridging agent to create interphase/interface chemical bonding between the ceramic and polymer phases, thereby providing a better dispersion of the two components and reducing the air voids that otherwise tend to form in and around micro-particle clusters [28]. Relatively large organic molecules of surfactant may provide reduced agglomeration of treated particles by steric hindrance effects. The polar units formed by side chains of the surfactant may also increase the affinity of the filler particles for the polymer matrix [29]. However, a surfactant with a very large molecular weight may hinder the dispersion of the ceramic particles because longer chains have lower mobility and become increasingly ineffective in inhibiting agglomeration [28,30]. For these reasons, octyl gallate ($M_{w}=282.33$ g/mol) was chosen as a surfactant, which has four side polar groups: three −OH groups that react with the surface of BaTiO_3_ and one C=O binding with C=C in the ABS chain.

Table 1 presents the dielectric characterisation of FDM 3D-printed BaTiO_3_/ABS composite coupons with various loadings of surfactant in the range of 0–1.5 wt % measured at 15 GHz. All the experiments were done repeatedly with three-to-five samples for each coupon. The average measured volume ratio of BaTiO_3_ was 32.6 vol %, which was approximately 8% greater than the nominal of 30 vol %. The relative dielectric permittivity increased with increasing surfactant fraction and reached ϵ=11.04 for coupon 3, containing 1 wt % octyl gallate (OG). However, further increases in OG fraction reduced the dielectric constant by ~10%, which may be caused by the increasing inhomogeneity in the composite microstructure (see below).

**Figure 1:**
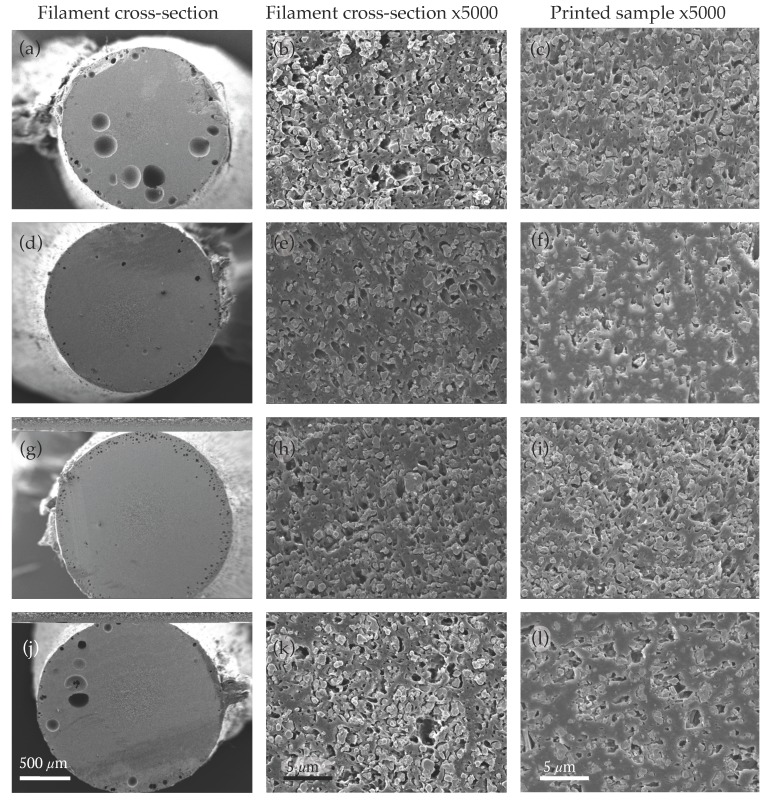
Electron micrographs of the cross-section of 30 vol % BaTiO_3_/acrylonitrile butadiene styrene (ABS) filaments (left and middle columns) and 3D-printed coupons (right column). (**a**–**c**) BaTiO_3_/ABS with 0 wt % surfactant; (**d**–**f**) 0.5 wt % surfactant; (**g**–**i**) 1.0 wt % surfactant; (**j**–**l**) 1.5 wt % surfactant.

Figure 1a,b shows low and high magnification SEM images of the BaTiO_3_/ABS filament cross-sections, taken from randomly chosen sections, without any surfactant. Micro-particles of BaTiO_3_ were generally not in close contact with the ABS, and the filament contained a considerable fraction of micro-pores (Figure 1b,c) and bubbles up to 200 μm in diameter (Figure 1a). Additionally, BaTiO_3_ micro-particles frequently formed irregularly-shaped and porous clusters.

Typical cross-section SEM images from filaments 2 to 4 (Figure 1d–l) show that the addition of surfactant was primarily to reduce voids, because BaTiO_3_ particles were more readily contacted and embedded more uniformly in the ABS, with less clustering, making it easier for polymer to fill the gaps between the particles. This reduction of voids in the FDM filaments correlated strongly with the increase of permittivity in the corresponding FDM printed coupons in Table 1. Coupon 4 with 1.5 wt % OG surfactant had a slightly lower dielectric permittivity due to an excess of surfactant, and the filler/matrix contact was undermined. Coupon 3 with 1 wt % of OG surfactant showed qualitatively the best micro-particle dispersion and the lowest porosity; it also showed a maximised dielectric permittivity and minimised tangent loss.

The 3D-printed coupons (right column in Figure 1) showed less porosity than the corresponding filament cross-section images (middle column in Figure 1). During the printing process, the filament was heated up to 245 °C (into the molten state of ABS), where small air bubbles and voids were mobile and fused together as the composite material was extruded through the heated nozzle. The trend of printed porosity was similar to that from the filament, with higher porosity in printed coupons 1 (Figure 1c) and 4 (Figure 1l) and lower porosity in coupons 2 (Figure 1f) and 3 (Figure 1i). Overall, coupons 2–4 (Figure 1f,i,l) showed qualitative improvements in the uniformity of BaTiO_3_ micro-particles.

### 2.2. Effect of Plasticiser

Plasticisers modify the properties of polymers, typically increasing elongation to failure and decreasing tensile strength by reducing intermolecular friction between polymer molecules [31]. In effect, when a relatively stiff plastic part is flexed, the polymer molecules move relative to each other either elastically or plastically, and the plasticiser acts to lubricate the relative movement of the polymer micellae.

**Figure 2:**
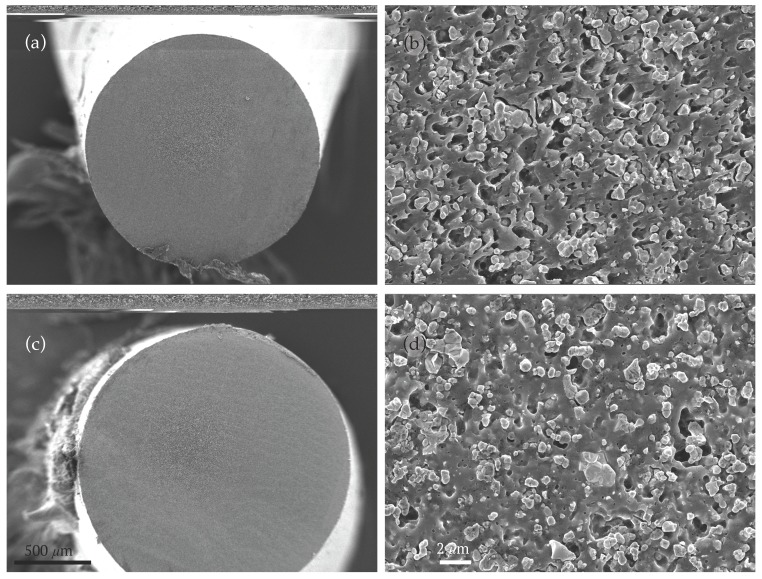
Electron micrographs of the cross-section of filaments. (**a**,**b**) Filament 5; (**c**,**d**) Filament 6.

To study the effect of plasticiser on the morphological, microstructural, and dielectric properties of the BaTiO_3_/ABS composites, two additional filaments (5 and 6) were fabricated, comprising the basic formulation of filament 3 (30 vol % BaTiO_3_/ABS with 1 wt % surfactant) with 5 wt % and 10 wt % dibutyl phthalate respectively. Figure 2 shows the resulting SEM cross-section images of the filament. During extrusion, the addition of plasticiser improved the ceramic–polymer composite flow through the extrusion die [32] and the filament surface roughness, while the BaTiO_3_ micro-particles dispersion remained relatively uniform.

**Figure 3:**
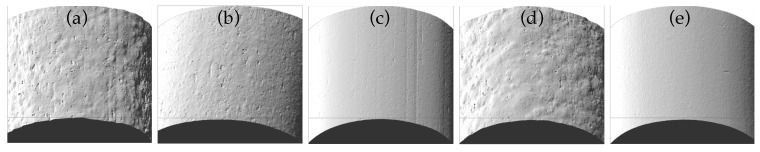
Optical images of the surface of composite filaments. (**a**) Filament 1 (roughness Ra=4.26 μm); (**b**) Filament 2 (Ra=1.49 μm); (**c**) Filament 3 (Ra=0.72 μm); (**d**) Filament 4 (Ra=4.01 μm); (**e**) Filament 5 (Ra=0.57 μm).

Figure 3 shows optical images of the surface of filaments 1 to 5. Filament 1 had the roughest surface, with an average roughness Ra=4.26 μm, while filament 5 had a roughness Ra=0.57 μm—similar to the original commercial ABS (Ra=0.55, not shown). Since all filaments were extruded from the same single screw extruder with the same 1.6 mm diameter nozzle, changes in the surface roughness of the filaments were ascribed to the different amounts of filament surfactant and plasticiser. Filament 4 (1.5 wt % surfactant) had a similar rough surface as filament 1, due to the greater agglomeration at excess surfactant fractions as previously shown in Figure 1k.

The addition of plasticiser also reduced the pressure of the extrusion [32], and the composite filament flowed faster from the nozzle for a constant pressure, and more reliably formed a continuous filament of smaller diameter. The plasticiser also decreased the ABS glass transition temperature $T_{g}$ from $T_{g}=102$ °C in filament 3 with no plasticiser to $T_{g}=53$ °C and $T_{g}=20$ °C for filaments 5 and 6, respectively (see Figure S1 in the Supplementary Materials). Note also that adding the OG surfactant also reduced $T_{g}$ (although less dramatically), suggesting that OG—as intended—was relatively soluble in ABS [33].

**Figure 4:**
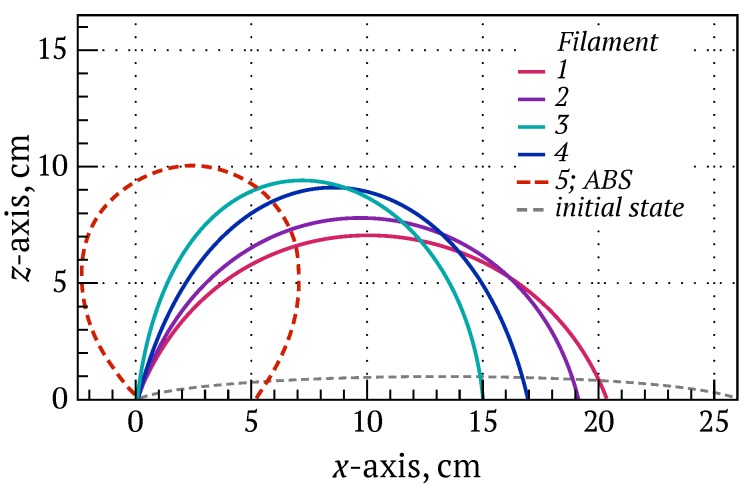
Typical bend test behavior of ABS and BaTiO_3_/ABS composite filaments. In each case, the left-hand side of the 26 cm filament length was stationary while the right-hand side gripping the filament along the *x*-axis slowly moved towards the left. The filament grips permitted only bending in the xz-plane. The curves describe the filament shape at the point of failure. The composite filament composed of 32 vol % BaTiO_3_/ABS with 1 wt % surfactant and 5 wt % plasticiser (filament 5) showed the same flexibility as pure ABS.

To investigate the effect of various additions on the filament mechanical properties, particularly filament ability to be coiled/uncoiled and fed robustly during 3D printing, a two-point filament bend test was devised (see Methods). The commercial ABS filament is typically supplied on a spool with radius of 4.5 cm, and provided the baseline against which the bend performance of the composite filaments could be assessed. The curvature of bending filaments just before they fractured was recorded from a calibrated video image during the bend test (Table 2), and Figure 4 reproduces the filament shape at this instant. Their sequence of increasing failure radius—namely, filaments 1, 2, 4 and 3—are in good agreement with tendency for filament pores and micro-particle clustering in the microstructures presented in Figure 1 and Figure 2.

Filament 5 composed of 32 vol % BaTiO_3_/ABS with 1 wt % surfactant and 5 wt % plasticiser had a similar failure radius of curvature to spool on which commercial ABS is supplied. A further increase in plasticiser fraction to 10 wt % produced a very soft filament that was not rigid enough to support itself when placed into the fixtures of the bend test (see Supplementary Materials); it could also not be FDM 3D-printed.

**Figure 5:**
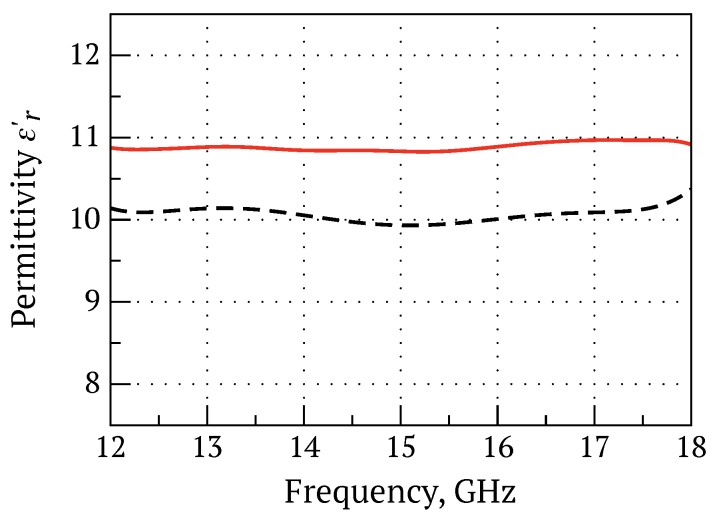
Measured real part of the relative dielectric permittivity as a function of frequency for 3D-printed coupons from filaments 5 (solid red) and 3 (dashed black).

The relative dielectric permittivity and loss of 3D-printed coupons using filament 5 were ϵ=11.0±0.195 and $\tan\delta =2.89\pm 0.12\times 10^{-2}$, measured at 15 GHz by the split-post dielectric resonator (SPDR) method. The relative dielectric permittivity of coupon 5 was approximately 0.063 lower than coupon 3, but the actual volume fraction *f* of BaTiO_3_ was less in coupon 5 (dielectric ratio ϵ/f=0.351 for coupon 5, and ϵ/f=0.341 for coupon 3). Figure 5 presents the real part of the relative dielectric permittivity ϵ as a function of frequency for 3D-printed coupons using filament 3 (nominal 30 vol % BaTiO_3_/ABS + 1 wt % surfactant) and filament 5 (same as filament 3 with added 5 wt % plasticiser) measured using a 7.89×15.79 mm^2^ rectangular waveguide and the well-known Nicholson–Ross–Weir retrieval method [34]. In the 12–18 GHz frequency range, the coupons exhibited relatively little dispersion in relative permittivity, and average dielectric permittivities of 10.85 and 10.10 were in good agreement with the SPDR measurements (Table 1).

### 2.3. Demonstration

**Figure 6:**
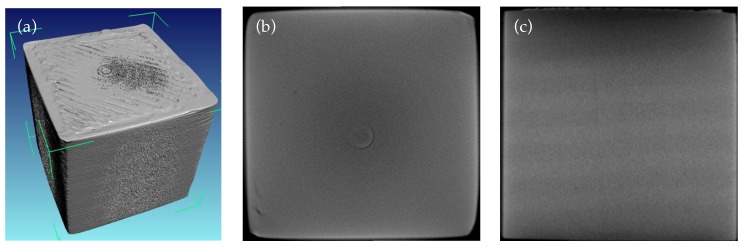
X-ray tomography image of the 3D-printed 15×15×15 mm^3^ cube using optimized composite filament 5. (**a**) Full 3D reconstruction; (**b**) xy-plane cut; (**c**) xz-plane cut. The images show the absence of voids or interlayer/interfilament porosity.

The 3D-printed coupons using the optimised filament comprising ~32 vol % BaTiO_3_/ABS composite with 1 wt % surfactant and 5 wt % plasticiser (filament 5) showed the highest dielectric permittivity of approximately 11, and had outstanding flexibility and a smooth surface. To demonstrate the printability of this optimised filament, several cube-like coupons with different printing precision and fabrication time were 3-D printed, as described in the Materials and Methods section. Due to the lower melting temperature of filament 5 (although it was not possible to determine this precisely), the print head temperature was decreased by 20 °C compared with pure ABS (230–245 °C). Figure 6a shows a reconstructed X-ray tomography image of a 15 mm side cube 3D-printed with 0.1 mm layer resolution and 100% infill using a RepRap FDM 3D printer. As seen from the plane cross-sections in Figure 6b,c, the printed internal structure was dense and with no resolvable voids or interlayer air gaps/porosity, as is otherwise often found in printed composites [6].

**Figure 7:**
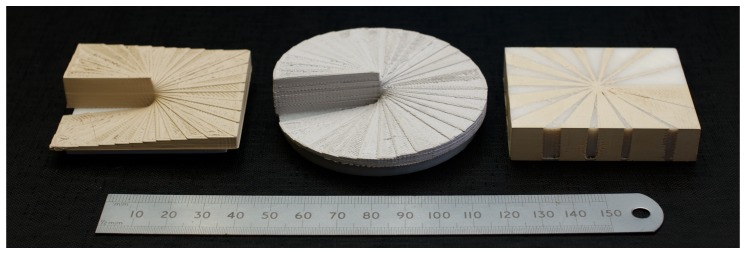
Demonstration of several 3D-printed microwave devices for the generation of orbital angular momentum.

Other examples of more device-like printed structures using filament 5 are presented in Figure 7. In each case, the devices are versions of a 3D-printed spiral phase plate, which is an optical device that imparts an azimuthal phase shift onto incident radiation. This azimuthal phase retardation can be achieved either by continuous variation in thickness, or by a local change in the material relative dielectric permittivity, and both types are shown. For changes in thickness (i.e., optical length), the total step height was chosen so that the total phase shift around the axis of the phase plate was 2π. The phase plates were printed with a 0.2 mm layer thickness and took approximately 3 h using commercial MakerBot Desktop software with standard settings.

## 3. Materials and Methods

High-permittivity filaments were fabricated based on the method described in [6]. Commercially-supplied acrylonitrile butadiene styrene (ABS MFI-22, Styrolution, Germany) was dissolved in acetone until total dissolution, and then 30 vol% micro-particles of barium titanate powder (Sigma Aldrich, Dorset, UK) were added and stirred for 12 h in order to achieve a uniform dispersion.

Various amounts (see Table 1) of octyl gallate (C_15_H_22_O_5_) and dibutyl phthalate (C_18_H_22_O_4_) (both Sigma Aldrich) used as surfactant and plasticiser, respectively, were added to alter the binding and mechanical properties of the BaTiO_3_/ABS composite. The suspension was spread out on a tray in a fume cupboard and then placed in an oven for 8 h at 70 °C to ensure the acetone had fully evaporated. The dried composite was then mechanically blended and extruded (at a temperature in the range 160–180 °C) through a desktop single screw extruder (Noztek, London, UK) with a 1.6 mm die orifice to form a continuous filament of approximately 1.75 mm in diameter. The coupons, cubes, and optical devices were printed using either a RepRap Mendel90 or MakerBot Replicator 2X desktop 3D printers using public domain software and standard operational conditions.

Cross-sections of the filaments and the printed coupons for electron microscopy were prepared using a rotary microtome diamond blade Reichert-Jung Ultracut (Leica Microsystems, UK). Scanning electron imaging was carried out using a JEOL JSM-6500F (JEOL Ltd.) electron microscope operated at 5 kV. Optical microscope images were obtained using a μsurf (NanoFocus AG, Germany) digital microscope with a × 10 lens. The surface roughnesses of the filaments were determined as $Ra=\frac{1}{L}\int_{0}^{L}\left|Z(x)\right|dx$, where *L* is the evaluation length and Z(x) is the profile height function; the surface roughness was calculated as the arithmetic average of a set of individual measurements.

Thermogravimetric analysis (TGA) of the filament feedstock in which the polymer fraction was fully volatilized at high temperature was used to measure the remaining weight fraction of inorganic particulates in the filaments, and was performed using a PerkinElmer thermogravimetric analyzer. Samples were heated from 40 °C to 600 °C at a heating rate of 10 °C/min, under a flowing air atmosphere. Differential scanning calorimetry (DSC) of the filament feedstock was performed using a PerkinElmer Diamond DSC with alumina as the reference, from 0 °C to 300 °C at a heating/cooling rate of 20 °C/min.

The complex dielectric properties of 3D-printed coupons were obtained using a Rohde & Schwarz ZNB20 vector network analyser (Rohde & Schwarz UK Ltd., Fleet, UK) and split-post dielectric resonator (SPDR) (QWED, Warsaw, Poland) and a Ku-band waveguide technique. The SPDR is designed for a nominal 15 GHz frequency and the actual measurements were taken at a frequency close to nominal.

Filament bend testing was performed using an in-house designed rig. Briefly, the two ends of filament length of 26.5±0.5 cm were clamped in holders free to rotate in the vertical xz-plane only. While one holder was fixed at x=0 cm, the other end slid smoothly along a rail with a constant velocity of 4.7 cm/s toward the origin, starting from x=26 cm, so that the filament was progressively bent in the vertical xz-plane. The process was digitally videoed normal to the xz-plane and the filament curvature at the point of failure retrieved. The curvature was calculated by best fit of the filament shape to a dome where the radius of curvature was calculated from $R=[{\left(d/2\right)}^{2}+h^{2}]/2h$, where *d* is the diameter of the dome and *h* is the height from the base to the highest point in the dome.

## 4. Conclusions

An optimised formulation of a high relative dielectric permittivity, low loss BaTiO_3_/ABS composite filament for FDM 3D printing has been demonstrated. The optimised filament had a commercial quality surface finish and an effective relative dielectric permittivity $\epsilon_{r}=11$ in printed coupons, which was typically 30% to 40% higher than previously reported for a similar composite system. Critical to the printability and dielectric performance was the use of optimised concentrations of surfactant (1 wt %) and plasticiser (5 wt %), which facilitated an acceptably uniform micro-particle distribution and reduction of void fraction, and enhanced elastic/plastic properties. Several 3D-printed structures were fabricated and demonstrated the applicability of the bespoke filament for robust long-time printing without interruption. These types of composites with enhanced and spatially controlled electromagnetic properties will widen the application range of 3D printed parts in the radio communication sector, including direct 3D-printing of antennas, microwave horns, and compact lenses.

## Figures and Tables

**Table 1:** Dielectric permittivity of 3D-printed nominal 30 vol % BaTiO_3_BaTiO3/ABS composites at 15 GHz with various loadings of surfactant; the measured BaTiO_3_ fraction was obtained by thermogravimetric analysis (TGA).

Coupon	Surfactant wt %	Measured BaTiO_3_ vol %	Permittivity $\epsilon_{r}^{\prime }$	Loss $\tan\delta \times 10^{-2}$
1	0	33.2	10.68 ± 0.50	3.25 ± 0.4
2	0.5	32.6	10.88 ± 0.35	2.05 ± 0.1
3	1.0	32.4	11.04 ± 0.21	3.03 ± 0.6
4	1.5	32.7	10.08 ± 0.16	3.43 ± 0.1

**Table 2:** Bending filament curvature at failure.

Filament	Distance Travelled, cm	Curvature, cm
ABS	21.0	4.5
1	5.8 ± 0.10	10.8 ± 0.2
2	7.0 ± 0.35	9.7 ± 0.9
3	11 ± 1.5	7.7 ± 2.3
4	9 ± 0.5	8.5 ± 1.4
5	21.0	4.5

## Supplementary Materials

The following are available online at www.mdpi.com/1996-1944/10/10/1218/s1: Figure S1: Differential scanning calorimetry measurements in filaments. Video S1: Bend testing of composite filaments in comparison to commercial ABS.

## References

[1] Quevedo-Teruel O., Tang W., Mitchell-Thomas R.C., Dyke A., Dyke H., Zhang L., Haq S., Hao Y. (2013). Transformation optics for antennas: Why limit the bandwidth with metamaterials?. Sci. Rep..

[2] Isakov D., Stevens C.J., Castles F., Grant P.S. (2017). 3D-Printed High Dielectric Contrast Gradient Index Flat Lens for a Directive Antenna with Reduced Dimensions. Adv. Mater. Technol..

[3] Isakov D.V., Lei Q., Castles F., Stevens C.J., Grovenor C.R.M., Grant P.S. (2016). 3D printed anisotropic dielectric composite with meta-material features. Mater. Des..

[4] Haring A.P., Khan A.U., Liu G., Johnson B.N. (2017). 3D Printed Functionally Graded Plasmonic Constructs. Adv. Opt. Mater..

[5] Boyle B.M., French T.A., Pearson R.M., McCarthy B.G., Miyake G.M. (2017). Structural Color for Additive Manufacturing: 3D-Printed Photonic Crystals from Block Copolymers. ACS Nano.

[6] Castles F., Isakov D., Lui A., Lei Q., Dancer C.E.J., Wang Y., Janurudin J.M., Speller S.C., Grovenor C.R.M., Grant P.S. (2016). Microwave dielectric characterisation of 3D-printed BaTiO_3_/ABS polymer composites. Sci. Rep..

[7] Grant P.S., Castles F., Lei Q., Wang Y., Janurudin J.M., Isakov D., Speller S., Dancer C., Grovenor C.R.M. (2015). Manufacture of electrical and magnetic graded and anisotropic materials for novel manipulations of microwaves. Philos. Trans. R. Soc. A.

[8] Leigh S.J., Purssell C.P., Bowen J., Hutchins D.A., Covington J.A., Billson D.R. (2011). A miniature flow sensor fabricated by micro-stereolithography employing a magnetite/acrylic nanocomposite resin. Sens. Actuator A Phys..

[9] Czyzewski J., Burzyński P., Gawel K., Meisner J. (2009). Rapid prototyping of electrically conductive components using 3D printing technology. J. Mater. Proc. Technol..

[10] Leigh S.J., Bradley R.J., Purssell C.P., Billson D.R., Hutchins D.A. (2012). A simple, low-cost conductive composite material for 3D printing of electronic sensors. PLoS ONE.

[11] Le Duigou A., Castro M., Bevan R., Martin N. (2016). 3D printing of wood fibre biocomposites: From mechanical to actuation functionality. Mater. Des..

[12] Lopes A.J., MacDonald E., Wicker R.B. (2012). Integrating stereolithography and direct print technologies for 3D structural electronics fabrication. Rapid Prototyp. J..

[13] Woodward D.I., Purssell C.P., Billson D.R., Hutchins D.A., Leigh S.J. (2015). Additively manufactured piezoelectric devices. Phys. Status Solidi A.

[14] Yang Y., Chen Z., Song X., Zhu B., Hsiai T., Wu P.-I., Xiong R., Shi J., Chen Y., Zhou Q. (2016). Three dimensional printing of high dielectric capacitor using projection based stereolithography method. Nano Energy.

[15] Kurimoto M., Yamashita Y., Ozaki H., Kato T., Funabashi T., Suzuoki Y. 3D printing of conical insulating spacer using alumina/UV-cured-resin composite. Proceedings of the 2015 IEEE Conference on Electrical Insulation and Dielectric Phenomena (CEIDP).

[16] Leigh S.J., Purssell C.P., Billson D.R., Hutchins D.A. (2014). Using a magnetite/thermoplastic composite in 3D printing of direct replacements for commercially available flow sensors. Smart Mater. Struct..

[17] Nikzad M., Masood S.H., Sbarski I. (2011). Thermo-mechanical properties of a highly filled polymeric composites for Fused Deposition Modeling. Mater. Des..

[18] Shemelya C.M., Rivera A., Perez A.T., Rocha C., Liang M., Yu X., Kief C., Alexander D., Stegeman J., Xin H. (2015). Mechanical, Electromagnetic, and X-ray Shielding Characterization of a 3D Printable Tungsten—Polycarbonate Polymer Matrix Composite for Space-Based Applications. J. Electron. Mater..

[19] Zhong W., Li F., Zhang Z., Song L., Li Z. (2001). Short fiber reinforced composites for fused deposition modeling. Mater. Sci. Eng. A.

[20] Ning F., Cong W., Qiu J., Wei J., Wang S. (2015). Additive manufacturing of carbon fiber reinforced thermoplastic composites using fused deposition modeling. Compos. Part B Eng..

[21] Mariappan B., Jaisankar S.N. (2017). Properties of polyurethane nanocomposite filaments for conductive textile applications. J. Thermoplast. Compos. Mater..

[22] Gray R.W., Baird D.G., Bohn J.H. (1998). Thermoplastic composites reinforced with long fiber thermotropic liquid crystalline polymers for fused deposition modeling. Polym. Compos..

[23] Kokkinis D., Schaffner M., Studart A.R. (2015). Multimaterial magnetically assisted 3D printing of composite materials. Nat. Commun..

[24] Shemelya C., Rosa A., Torrado A.R., Yue K., Domanowskie J., Bonacusee P.J., Martin R.E., Juhasz M., Hurwitz F., Wicker R.B. (2017). Anisotropy of thermal conductivity in 3D printed polymer matrix composites for space based cube satellites. Addit. Manuf..

[25] Murphy C.A., Collins M.N. (2016). Microcrystalline cellulose reinforced polylactic acid biocomposite filaments for 3D printing. Polym. Compos..

[26] RepRap. http://reprap.org.

[27] Curecheriu L., Balmus S.-B., Buscaglia M.T., Buscaglia V., Ianculescu A., Mitoseriu L. (2012). Grain Size-Dependent Properties of Dense Nanocrystalline Barium Titanate Ceramics. J. Am. Ceram. Soc..

[28] Zhou T., Zha J.-W., Cui R.-Y., Fan B.-H., Yuan J.-K., Dang Z.-M. (2011). Improving dielectric properties of BaTiO_3_/ferroelectric polymer composites by employing surface hydroxylated BaTiO_3_ nanoparticles. ACS Appl. Mater. Interfaces.

[29] Ogitani S., Bidstrup-Allen S.A., Kohl P.A. (2000). Factors influencing the permittivity of polymer/ceramic composites for embedded capacitors. IEEE Trans. Adv. Packag..

[30] Ramajo L., Castro M.S., Reboredo M.M. (2007). Effect of silane as coupling agent on the dielectric properties of BaTiO_3_-epoxy composites. Compos. Part A Appl. Sci. Manuf..

[31] Kirkpatrick A. (1940). Some Relations Between Molecular Structure and Plasticizing Effect. J. Appl. Phys..

[32] Tylkowski B., Marturano V., Cerruti P., Ambrogi V. (2017). Polymer additives. Phys. Sci. Rev..

[33] Wood L.A. (1958). Glass transition temperatures of copolymers. J. Polym. Sci..

[34] Nicholson A.M., Ross G.F. (1970). Measurement of the intrinsic properties of materials by time-domain techniques. IEEE Trans. Instrum. Meas..

