# Supplementary material for: Fabrication of Composite Filaments with High Dielectric Permittivity for Fused Deposition 3D Printing
Source: Materials (Basel). 2017 Oct 23;10(10):1218. doi: 10.3390/ma10101218 (PMC5667024; doi:10.3390/ma10101218)
Supplement: Supplementary file 1 [file materials-10-01218-s001.zip › materials-234299-supplementary/SupplementaryMaterials.pdf]

Article

Fabrication of Composite Filaments with High Dielectric Permittivity for Fused Deposition 3D Printing

Yingwei Wu, Dmitry Isakov * and Patrick S. Grant

University of Oxford, Department of Materials, Parks road, OX1 3PH Oxford, UK

* Correspondence: dmitry.isakov@materials.ox.ac.uk

Academic Editor: name

Version September 30, 2017 submitted to Materials

Supplementary materials

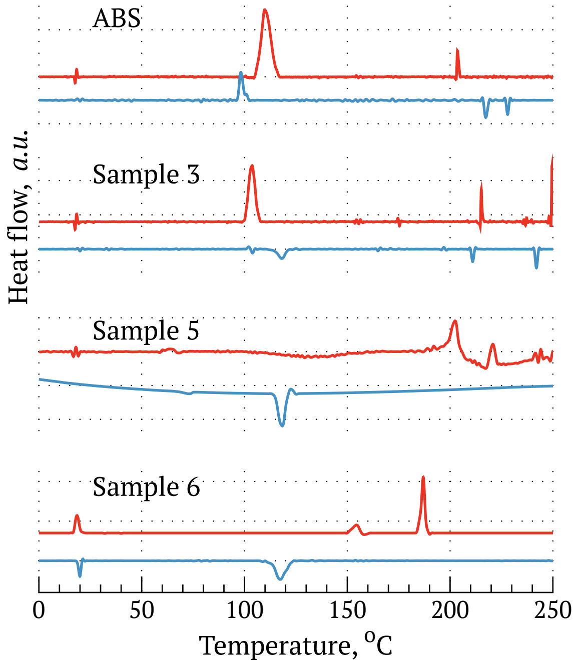

Figure S1. Differential scanning calorimetry measurements of heating (red curves) and cooling (blue curves) runs in filaments. **(a)** ABS; **(b)** Sample 3; **(c)** Sample 5; **(d)** Sample 6.

Video S1: Bend test of bespoke filaments in comparison to commercial ABS.

From the video, the breaking sequence of the filament is ranked as Sample 1, Sample 2, Sample4, and lastly sample 3. Sample 5 and standard ABS don't break. Sample 6 is not rigid enough to support its own weight, as can be seen that the filament is hanging between the fixtures and doesn't form a curve when the unfixed end is moving. The breaking sequence also corresponds to the dielectric measurement values as well as the SEM images. All these indicate that sample 5 has the optimal combination of surfactant and plasticiser and can provide both dielectric properties as well as flexibility.

© 2017 by the authors. Submitted to *Materials* for possible open access publication under the terms and conditions of the Creative Commons Attribution (CC BY) license (http://creativecommons.org/licenses/by/4.0/).
